# Sulfated peptides: key players in plant development, growth, and stress responses

**DOI:** 10.3389/fpls.2024.1474111

**Published:** 2024-10-22

**Authors:** Penghong Zhang, Jiangzhe Zhao, Wei Zhang, Yongfeng Guo, Kewei Zhang

**Affiliations:** ^1^ Zhejiang Provincial Key Laboratory of Biotechnology on Specialty Economic Plants, College of Life Sciences, Zhejiang Normal University, Jinhua, Zhejiang, China; ^2^ Tobacco Research Institute, Chinese Academy of Agricultural Sciences, Qingdao, Shandong, China

**Keywords:** sulfated peptide, phytohormone, plant growth and development, environmental signals, stress response, immunity

## Abstract

Peptide hormones regulate plant development, growth, and stress responses. Sulfated peptides represent a class of proteins that undergo posttranslational modification by tyrosylprotein sulfotransferase (TPST), followed by specific enzymatic cleavage to generate mature peptides. This process contributes to the formation of various bioactive peptides, including PSKs (PHYTOSULFOKINEs), PSYs (PLANT PEPTIDE CONTAINING SULFATED TYROSINE), CIFs (CASPARIAN STRIP INTEGRITY FACTOR), and RGFs (ROOT MERISTEM GROWTH FACTOR). In the past three decades, significant progress has been made in understanding the molecular mechanisms of sulfated peptides that regulate plant development, growth, and stress responses. In this review, we explore the sequence properties of precursors, posttranslational modifications, peptide receptors, and signal transduction pathways of the sulfated peptides, analyzing their functions in plants. The cross-talk between PSK/RGF peptides and other phytohormones, such as brassinosteroids, auxin, salicylic acid, abscisic acid, gibberellins, ethylene, and jasmonic acid, is also described. The significance of sulfated peptides in crops and their potential application for enhancing crop productivity are discussed, along with future research directions in the study of sulfated peptides.

## Introduction

Secretory peptides are widely distributed in various plant species, including monocots such as wheat, rice, and asparagus, and dicots such as *Arabidopsis*, soybeans, and carrots (Tian et al., 2022). These peptides play crucial roles in multiple aspects of plant growth and development, as well as immune responses. However, the secretion and modification mechanisms of these peptides remain largely elusive due to their diverse structural characteristics.

Tyrosylprotein sulfotransferase (TPST) catalyzes the transfer of sulfate from 3′-phosphate-5′-phosphosulfate (PAPS) to the tyrosine in different proteins or peptides, which then facilitates the sulfation modification process (Moore, 2003; Kanan and Al-Ubaidi, 2013). This posttranslational modification plays a significant role in plants. In *Arabidopsis*, TPST modifies PSK, PSY, RGF, and CIF peptides into their active forms (Matsuzaki et al., 2010). These active forms have conserved functions across different plant species by regulating plant growth, development, and stress responses.

**Figure 1:**
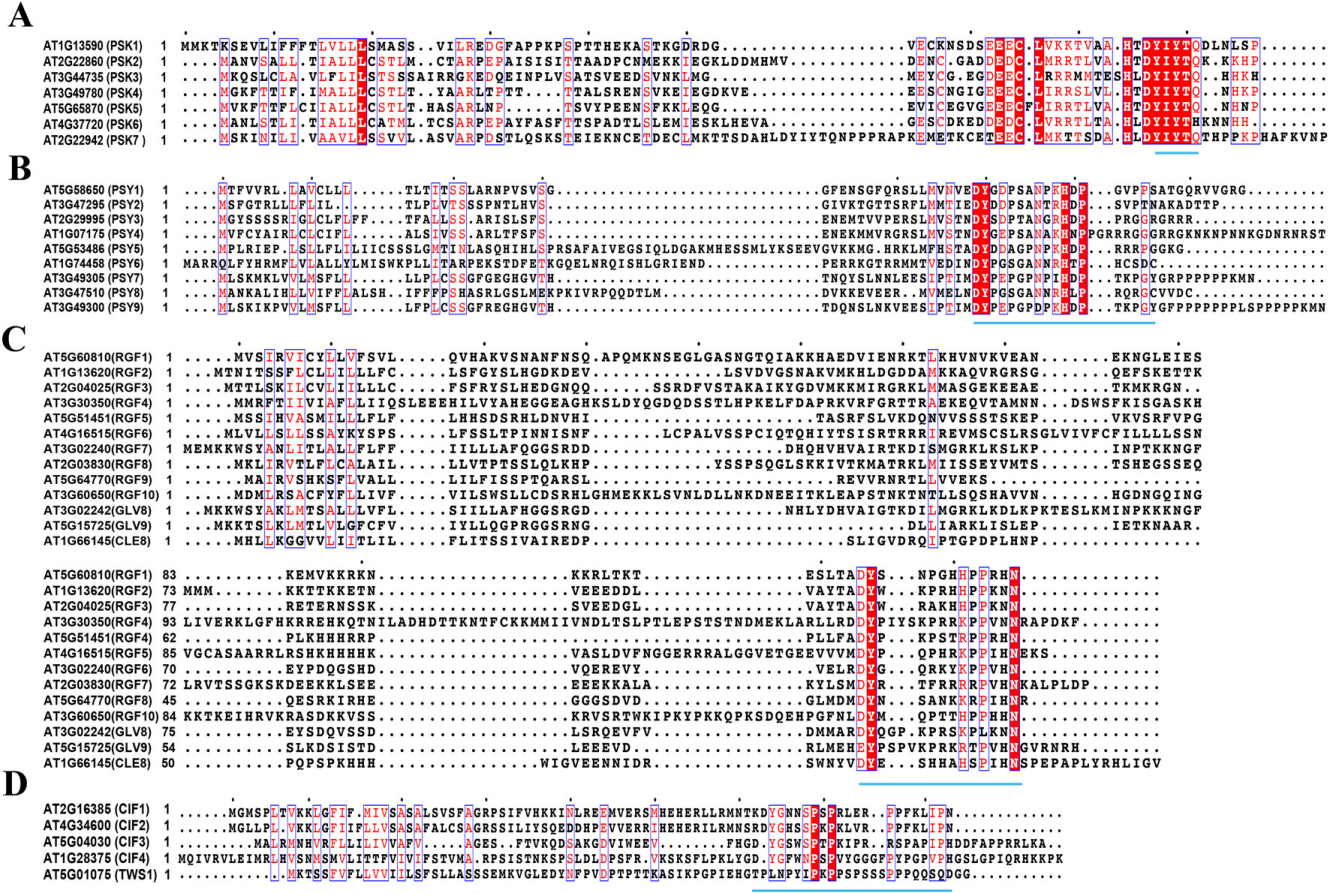
Amino acid sequences for peptide precursors (MUSCLE) **(A)**. Amino acid sequence of PSK precursors, **(B)**. Amino acid sequence of PSY precursors, **(C)**. Amino acid sequence of RGF precursors, **(D)**. Amino acid sequence of CIF precursors. The same amino acid residues are highlighted in red and white letters, and similar amino acid residues are highlighted in red letters. These amino acid residues are marked with blue box lines. Motifs responsible for encoding mature peptides are represented by blue underscores. The protein sequences for peptide precursors are from the National Center for Biotechnology Information (NCBI) and the Arabidopsis Information Resource (TAIR, https://www.arabidopsis.org) and are compared via the MUSCLE algorithm of MEGA software (Stecher et al., 2020), and then analyzed with ESPript 3.0 (https://espript.ibcp.fr/ESPript/cgi-bin/ESPript.cgi) (Robert and Gouet, 2014) to label the motifs in reported precursors. This sequence alignment result was modified from Matsubayashi, 2018 and Matsubayashi, 2011.

Here, we summarize the precursor amino acid sequences of four sulfated peptides that undergo posttranslational modifications by TPST and proteolysis to produce active peptides (**Figure 1**). We also provide insights into their functions and regulatory mechanisms by summarizing other previous studies that elucidated their precursor genes, receptors, and downstream signaling components. Together, this investigation concerning the functions and regulatory mechanisms of sulfated peptides is of great importance to understand plant growth regulation, stress responses, and their adaptability to diverse environmental conditions.

## TPST

Peptide sulfation of tyrosine exists in both animals and plants; however, the amino acid sequences of TPST differ significantly between these two kingdoms (Ouyang et al., 1998; Moore, 2003). In humans, TPSTs are classified as type II transmembrane proteins with a short N-terminal cytoplasmic domain, and their active sites reside within the *cis*-Golgi lumen (Ouyang et al., 1998; Moore, 2003). However, in *Arabidopsis*, TPST is categorized as a type I transmembrane protein with a C-terminal transmembrane domain (Komori et al., 2009).

*Arabidopsis* TPST functions as a single enzyme responsible for peptide sulfation, and its mutant has been shown to possess various pleiotropic phenotypes, such as growth retardation, abnormal pollen tubes and grain development, decreased primary root length accompanied by increased lateral density, aberrant vascular development, and impaired Casparian strip formation (Komori et al., 2009; Stuhrwohldt et al., 2015; Doblas et al., 2017; Nakayama et al., 2017; Holzwart et al., 2018; Kaufmann et al., 2021; Truskina et al., 2022).

These phenotypic alterations have been closely associated with four families of peptides: RGFs, PSKs, PSYs, and CIFs (Kaufmann and Sauter, 2019). Furthermore, XAPs (XYLEM SAP ASSOCIATED PEPTIDEs), a group of uncharacterized sulfated peptides identified from the xylem sap samples of soybean (*Glycine max*) and Medicago (*Medicago truncatula*), may serve as long-distance signals that regulate various plant developmental processes (Okamoto et al., 2015; Patel et al., 2018). In this review, however, we mainly focus on the functions of these four families of sulfated peptides, namely, PSKs, PSYs, RGFs, and CIFs, which have been extensively studied in plants.

## PSKs

### The precursors of PSK peptides

PSK peptides consist of five amino acids, including two sulfated tyrosines (Matsubayashi et al., 1996; Matsubayashi and Sakagami, 1996). Previously, it has also been shown that seven genes encode for PSK precursors and contain 87–109 amino acids in *Arabidopsis* (Kaufmann and Sauter, 2019) (**Table 1**).

**Table 1:** Structural features of tyrosine-sulfate modified small-peptide^a^.

AGI code	Name	Size of Precursor (aa)	Peptide Motif	Reference
AT1G13590	PSK1	87	YIYTQ	Matsubayashi and Sakagami, 1996; Kaufmann and Sauter, 2019
AT2G22860	PSK2	87	YIYTQ	Matsubayashi and Sakagami, 1996; Kaufmann and Sauter, 2019
AT3G44735	PSK3	81	YIYTQ	Matsubayashi and Sakagami, 1996; Kaufmann and Sauter, 2019
AT3G49780	PSK4	79	YIYTQ	Matsubayashi and Sakagami, 1996; Kaufmann and Sauter, 2019
AT5G65870	PSK5	77	YIYTQ	Matsubayashi and Sakagami, 1996; Kaufmann and Sauter, 2019
AT4G37720	PSK6	87	YIYTH	Matsubayashi and Sakagami, 1996; Kaufmann and Sauter, 2019
AT2G22942	PSK7	109	YIYTQ	Matsubayashi and Sakagami, 1996; Kaufmann and Sauter, 2019; Stuhrwohldt et al., 2021
AT5G58650	PSY1	75	DYGDPSANPKHDPGVPPS	Amano et al., 2007; Tost et al., 2021
AT3G47295	PSY2	71	DYDDPSANTRHDPSVPT	Tost et al., 2021; Ogawa-Ohnishi et al., 2022
AT2G29995	PSY3	71	DYSDPTANGRHDPPR	Tost et al., 2021; Ogawa-Ohnishi et al., 2022
AT1G07175	PSY4	87	DYGEPSANAKHNP	Tost et al., 2021; Ogawa-Ohnishi et al., 2022
AT5G53486	PSY5	104	DYDDAGPNPKHDPRRRPGGKG	Tost et al., 2021; Ogawa-Ohnishi et al., 2022
AT1G74458	PSY6	94	DYPGSGANNRHTPH	Tost et al., 2021; Ogawa-Ohnishi et al., 2022
AT3G49305	PSY7	78	DYPEPGPNPIHDP	Tost et al., 2021; Ogawa-Ohnishi et al., 2022
AT3G47510	PSY8	85	DYPGSGANNRHLPR	Tost et al., 2021; Ogawa-Ohnishi et al., 2022
AT3G49300	PSY9	86	DYPEPGPDPKHDP	Ogawa-Ohnishi et al., 2022
AT5G60810	RGF1/GLV11/CLEL8	116	DYSNPGHHPPRHN	Matsuzaki et al., 2010; Meng et al., 2012; Whitford et al., 2012
AT1G13620	RGF2/GLV5/CLEL1	109	DYWKPRHHPPKNN	Matsuzaki et al., 2010; Meng et al., 2012; Whitford et al., 2012
AT2G04025	RGF3/GLV7/CLEL3	110	DYWRAKHHPPKNN	Matsuzaki et al., 2010; Meng et al., 2012; Whitford et al., 2012; Li et al., 2020
AT3G30350	RGF4/GLV3	180	DYPIYSKPRRKPPVNN	Matsuzaki et al., 2010; Whitford et al., 2012
AT5G51451	RGF5/GLV10/CLEL7	88	DYPKPSTRPPRHN	Matsuzaki et al., 2010; Meng et al., 2012; Whitford et al., 2012
AT4G16515	RGF6/GLV1/CLEL6	147	DYPQPHRKPPIHN	Matsuzaki et al., 2010; Meng et al., 2012; Whitford et al., 2012
AT3G02240	RGF7/GLV4/CLEL4	102	DYGQRKYKPPVHN	Matsuzaki et al., 2010; Meng et al., 2012; Whitford et al., 2012
AT2G03830	RGF8/GLV6/CLEL2	123	DYRTFRRRRPVHN	Matsuzaki et al., 2010; Meng et al., 2012; Whitford et al., 2012
AT5G64770	RGF9/GLV2/CLEL9	79	DYNSANKKRPIHN	Matsuzaki et al., 2010; Meng et al., 2012; Whitford et al., 2012
AT3G60650	RGF10	143	DYMQPTTHPPHHN	Shinohara, 2021
AT3G02242	GLV8/CLEL5	111	DYQGPKPRSKPLKNN	Meng et al., 2012; Whitford et al., 2012
AT5G15725	GLV9	95	EYPSPVKPRKRTPVHN	Whitford et al., 2012
AT1G66145	CLE18	101	DYESHHAHSPIHN	Meng et al., 2012
AT2G16385	CIF1	83	DYGNNSPSPRLERPPFKLIPN	Doll et al., 2020; Fujita, 2021
AT4G34600	CIF2	83	DYGHSSPKPKLVRPPFKLIPN	Doll et al., 2020; Fujita, 2021
AT5G04030	CIF3	76	DYGSWSPTPKIPRRSPAPIPH	Doll et al., 2020; Fujita, 2021
AT1G28375	CIF4	102	DYGFWNPSPVYGGGFPYPGPVPH	Doll et al., 2020; Fujita, 2021
AT5G01075	TWS1	81	DYNFPVDPTPTTKASIKPGPIEH	Doll et al., 2020; Fujita, 2021

a The peptide motif in PSY4, PSY7 and PSY9 displays the conserved PSY motif.

Through RT-PCR and GUS staining analyses, it was found that the transcriptional expression of different PSK precursors varied in various tissues or organs. Notably, *PSK2* expression was only detected in pollens (Stuhrwohldt et al., 2015). However, the promoter activity of *PSK3* was detected in flower tips and whole pistils (excluding stigma and pedicel), but not in ovules or pollen (Stuhrwohldt et al., 2015). Additionally, *PSK4* expression can be detected in fertilized ovules and pistils (Stuhrwohldt et al., 2015). Furthermore, *PSK5* transcripts were detected in the vascular structure of both the filament and style of *Arabidopsis* (Stuhrwohldt et al., 2015).

Interestingly, *AtPSK3* and *AtPSK5* were induced by wounding; however, no similar pattern was observed for the precursors of *PSK1*, *PSK2*, or *PSK4* (Loivamaki et al., 2010). Transcriptome deep sequencing (RNA-seq) data revealed significantly low expression levels of *PSK6* in plants (Cheng et al., 2017). Moreover, *PSK6* encodes the PSK-related sequence YIYTH, which differs from PSK1–PSK5 by having a conserved pentapeptide sequence, YIYTQ, at the C-terminus (Kaufmann and Sauter, 2019) (**Table 1**). Current understanding suggests that *PSK6* may be a pseudogene (Kaufmann and Sauter, 2019). In addition to these precursors, AT2G22942 has also recently been identified as a putative PSK precursor gene from *Arabidopsis* genome annotation and contains two typical PSK (YIYTQ) sequences within its protein (Kaufmann and Sauter, 2019).

In summary, the unique effects of PSK precursors on plant growth and development may be attributed to their differential expression sites. Therefore, it is essential for researchers to analyze the functions associated with different PSK precursors to decipher how plants adapt to adverse environmental conditions.

### Types and active forms of PSKs

Sulfated pentapeptide PSK-α was characterized in plant growth that is capable of promoting cell proliferation in asparagus (*Asparagus officinalis L.*) and rice (*Oryza sativa L.*) at the nanomolar level (Matsubayashi et al., 1996, 1997). Previously, the mitogenic activity of asparagus cells in a conditioned medium (CM) aided with the identification of two factors: 1) sulfated pentapeptide PSK-α (H-Tyr(SO_3_H)-Ile-Tyr(SO_3_H)-Thr-Gln-OH) and 2) sulfated tetrapeptide PSK-β (H-Tyr(SO_3_H)-Ile-Tyr(SO_3_H)-Thr-OH) (Matsubayashi and Sakagami, 1996). These peptides exert their biological functions through tyrosine residue sulfation (Matsubayashi and Sakagami, 1996). Previous studies have shown that PSK-α activity is 10 times higher than that of PSK-β (Yamakawa et al., 1998b) and that PSK-β is an enzymatic degradation product of PSK-α (Yang et al., 1999). Therefore, researchers widely use PSK-α as the main active form.

Three additional PSK-like peptides, namely, PSK-γ (Y_SO3_VY_SO3_TQ), PSK-δ (Y_SO3_IY_SO3_TN), and PSK-ϵ (Y_SO3_VY_SO3_TN), were also discovered in legume species (Yu et al., 2019; Di et al., 2022; Yu et al., 2022). The precursor gene *GmPSKγ1*, which encodes the PSK-γ peptide, is primarily expressed in soybean (*Glycine max*) seeds (Yu et al., 2019). From previous studies, it was shown that the overexpression of this gene significantly increases the size and weight of *Arabidopsis* seeds by inducing embryo cell expansion, but it does not affect the activation of immune response (Liu et al., 2019; Yu et al., 2019). However, both PSK-δ and PSK-ϵ peptides increase the number of nodules and promote plant growth (Di et al., 2022; Yu et al., 2022), which suggests their potential roles enhance crop yield.

Proteolysis is crucial in mature peptide formation (Yang et al., 1999, 2001), and SBT1.1 is a subtilisin-like serine protease that induces callus proliferation by activating the proteolytic mechanism of the PSK4 precursor (Srivastava et al., 2008). SBT1.1 exhibits specificity in its proteolytic activity toward various PSK precursors, showing strong activity toward PSK4, low activity toward PSK2 and PSK5, and no activity toward PSK1, PSK3, and PSK6 (Srivastava et al., 2008). SBT3.8 exhibits C-terminal aspartic acid-dependent cleavage activity, enabling the generation of biologically active PSK peptides from the PSK1 precursor (Stuhrwohldt et al., 2021). These findings highlight the crucial role of specific proteins in the subtilisin gene family in mediating the production of bioactive PSK peptides and suggest that the additional members may be involved in processing to PSK precursors (Rautengarten et al., 2005). However, the precise mechanism by which subtilisin process PSK precursors remains to be further elucidated.

### The functions of PSKs in plant development and growth

Previous studies have shown that the PSK peptides exhibit multiple physiological functions in many plant species. For example, PSK can enhance cell proliferation both *in vitro* and *in vivo*, delay the etiolation rate of carrot cotyledons, promote elongation of cotton (*Gossypium hirsutum*) fiber cells, increase nodule formation in *Lotus japonicas* roots, stimulate tracheary element differentiation in Zinnia (*Zinnia elegans*), facilitate pollen germination and pollen tube elongation, as well as promote hypocotyl elongation and protoplast expansion in *Arabidopsis* (Yamakawa et al., 1998a; Matsubayashi et al., 1999; Yang et al., 1999; Chen et al., 2000; Stuhrwohldt et al., 2011; Han et al., 2014; Stuhrwohldt et al., 2015; Wang et al., 2015a). In the Chinese fir (*Cunninghamia lanceolata*), PSK also reduces peroxidase activity during the early stages of proembryo induction while also inducing the expression of proembryo-related genes, such as *WOX2*, thereby facilitating somatic embryo production (Hao et al., 2023). Additionally, PSK peptides activate fruit ripening pathways by promoting the phosphorylation of DEHYDRATION-RESPONSIVE ELEMENT BINDING PROTEIN 2F (SlDREB2F) at the 30th tyrosine site through interaction with the SlPSKR1 receptor kinase, thereby leading to tomato (*Solanum lycopersicum*) maturation and improved quality (Fang et al., 2024).

**Figure 2:**
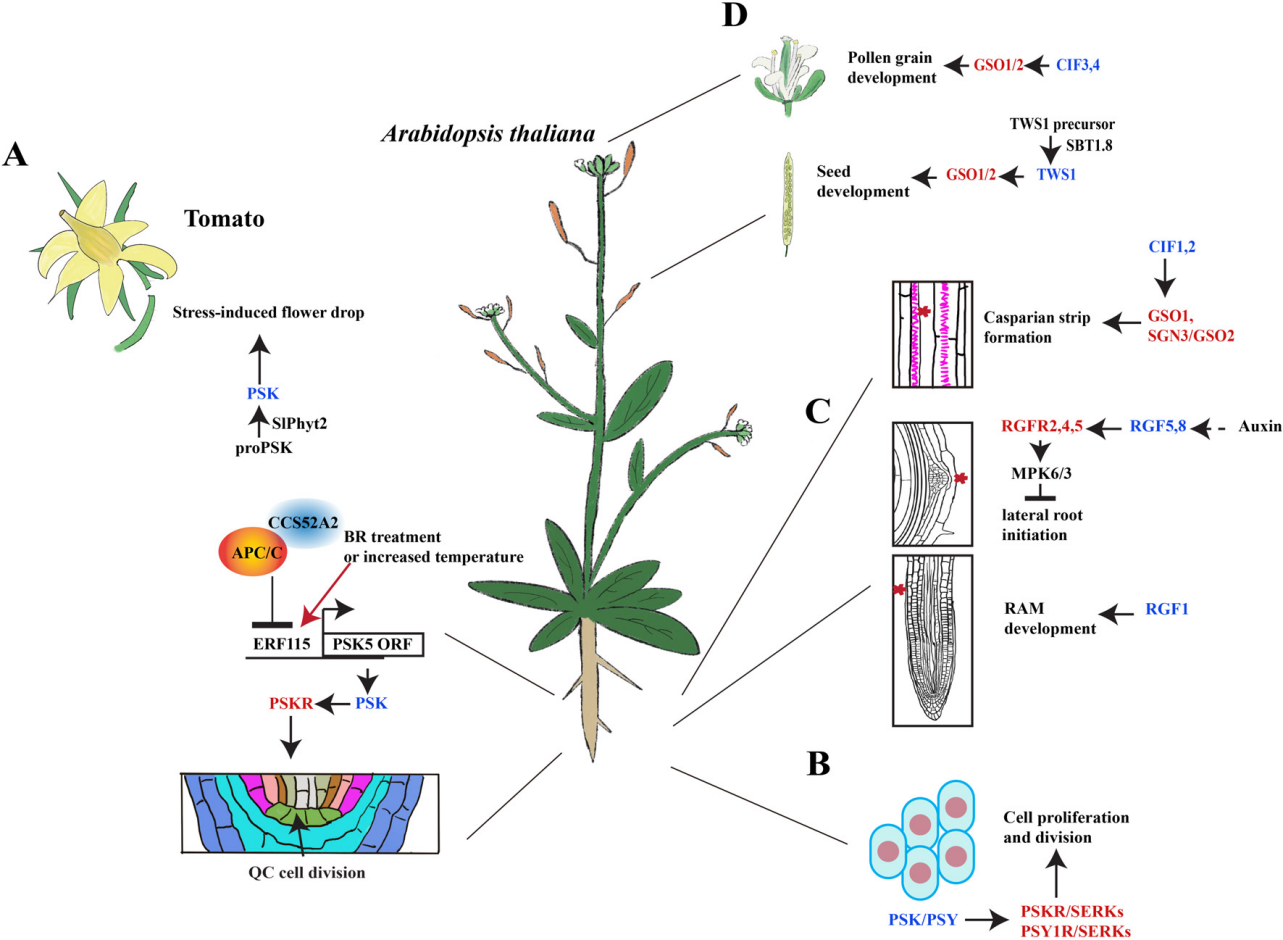
Signaling transduction of sulfated peptides during plant development. **(A)** PSK peptides induce the abscission of tomato floral organs and the division of the *Arabidopsis* quiescence center. BR, brassinosteroids; QC, quiescent center. **(B)** PSK and PSY peptides promote cell proliferation and division. **(C)** RGFs mediate the initiation of lateral roots and RAM development in *Arabidopsis*. RAM, root apical meristem. **(D)** CIF peptides promote the development of pollen, seeds, and Casparian strips in *Arabidopsis*. The schematic view in A was modified from Heyman et al., 2013 and Reichardt et al., 2020. Blue font represents peptides, whereas red font represents the peptide receptors.

Further studies suggest that the PSK peptide may interact with other hormones to regulate plant growth (**Table 2**). In *Arabidopsis*, PSK5 activates the APC/C^CCS52A2^ complex in the quiescent center (QC), inhibiting the accumulation of ERF115 transcription factors and downstream target gene expression, thereby suppressing root QC cell division (Heyman et al., 2013). Conversely, increased brassinosteroid (BR) levels and higher temperatures promote *ERF115* expression, leading to enhanced transcription of *PSK5* and activation of the mitotic cell cycle (Heyman et al., 2013) (**Figure 2A**). Overexpression of *PSK4* upregulates expansion-coding genes involved in cell wall loosening but reduces fertility rates (Yu et al., 2016).

**Table 2:** The interaction between sulfated peptides and other phytohormones.

Peptides	Gene	Descriptions	Species	Phytohormone	Functions	Reference
PSK	*PSK5*	Precursor	*Arabidopsis*	BR	Regulate the mitotic cell cycle; PSK-dependent growth	Hartmann et al., 2013; Heyman et al., 2013
	*PSK4*	Precursor	*Arabidopsis*	auxin	Plant growth and development	Badola et al., 2022
	*OsLPK1*	Receptor homolog	Rice	GA	Internodes elongation	Yang et al., 2013
	*OsPSKR15*	Receptor	Rice	ABA	Drought tolerance	Wang et al., 2015a
	*LjPSK*	Precursor	*L. japonicus*	JA	Rhizobia proliferation	Kanan and Al-Ubaidi, 2013
	*TPST*	Tyrosylprotein sulfotransferase	*Arabidopsis*	ethylene	Response to Cu deficiency	Wu et al., 2015
	*PSKR1*	Receptor	*Arabidopsis*	SA and JA	Antagonism to biotrophic and necrotic pathogens	Igarashi et al., 2012; Mosher et al., 2013; Song et al., 2023
	*OsPSKR1*	Receptor	Rice	SA	Leaf streak resistance	Yang et al., 2019
	*GbIncRNA7*	Precursor	Cotton	IAA	*Verticillium* dahlia resistance	Zhang et al., 2022
	*SlPSKR1*	Receptor	Tomato	auxin	*B. cinerea* resistance	Zhang et al., 2018
RGF4/6/9	*RGF4/6/9*	Precursor	*Arabidopsis*	auxin	Root geotropism	Whitford et al., 2012
RGF5/8	*RGFR2*/4/5	Receptor	*Arabidopsis*	auxin	Lateral root development	Fernandez et al., 2020
RGF9	*RGFR1*	Receptor	*Arabidopsis*	ethylene	PTI immune response	Stegmann et al., 2022

MicroRNAs (miRNAs) are endogenous non-coding small RNAs. miR858a is a type of miRNA involved in flavonoid biosynthesis and miPEP858a peptide formation in *Arabidopsis*. It is known to play an important regulatory role in plant growth regulation, development process, phenylpropanoid pathway, and response to stress (Sharma et al., 2016, 2020). Previously, Trivedi’s group observed that the overexpression of *PSK4* restores defective phenotypes from impaired growth and retardation in *miR858a* mutant plants, which indicates that PSK4 participates in regulating plant growth and development through the miR858a/miRPEP848a signaling pathway (Badola et al., 2022). Furthermore, MYB3 directly binds to both *PSK4* and *miR858a* promoters to regulate the expression of auxin-related genes such as *YUCs*, *PINs*, *AUX1*, and *ABCB19* (Badola et al., 2022).

Likewise, it has also been found that a homologous gene for the PSK receptor, OsLRK1, inhibits GA biosynthesis, which can suppress rice internodal elongation (Yang et al., 2013). Luo’s group determined that rice Nipponbare callus treated with PSK peptides downregulate *OsKO2* expression, which is involved in GA biosynthesis (Yang et al., 2013). Together, these results indicate a cross-talk between the PSK signaling pathway and GA biosynthesis in plant growth regulation (Yang et al., 2013).

Furthermore, the PSK receptor *OsPSKR15* in rice can be induced by ABA (Nagar et al., 2022). Inhibition of BR biosynthesis abolishes the responses of wild type and *tpst* and *PSKR1*-overexpressing plants to PSK peptides, suggesting that BR mediates PSK-dependent growth (Hartmann et al., 2013). Additionally, overexpression of *LjPSK1* or *AtPSKR1* can downregulate JA signal transduction while simultaneously increasing nodule numbers (Wang et al., 2015a). Under copper (Cu) deficiency conditions, root elongation in the *attpst* mutant exhibits increased sensitivity to Cu compared with the wild type; meanwhile, ethylene production is significantly higher in the mutant than that in the wild type (Wu et al., 2015). This phenotype can be rescued by adding the ethylene response inhibitor AgNO_3_ or treating with PSK peptides (Wu et al., 2015), indicating that TPST inhibits ethylene synthesis through the PSK peptide signaling pathway.

The BR receptor, BRASSINOSTEROID INSENSITIVE 1 (BRI1), is essential for maintaining normal growth of root vascular cells and the function of the receptor-like protein RLP44 (Wolf et al., 2014). Typically, RLP44 forms a complex with BRI1 and its coreceptor BAK1, playing a crucial role in suppressing the compensatory activation of the BR signaling pathway during pectin de-methylesterification in the cell wall (Wolf et al., 2014). Furthermore, RLP44 acts as a scaffold to stabilize PSKR1-BAK1 and BRI1-BAK1 complexes, thereby regulating xylem differentiation in a BRI1-dependent manner (Holzwart et al., 2018). Although phosphorylation-induced changes in RLP44 control its subcellular localization, the phosphorylation status does not affect its role in PSK signal transduction (Garnelo Gomez et al., 2021). Similar to the *rlp44* mutant, the *pskr1pskr2* double mutant exhibits an increased metaxylem cell number; however, the exogenous addition of PSK peptides restores the *rlp44* phenotype (Holzwart et al., 2018), which indicates that RLP44 promotes PSK signaling transduction within the vascular system that in turn inhibits procambium-to-xylem transition. Nevertheless, further investigation is required to elucidate the influence of PSK signaling on xylem differentiation and identify potential downstream targets.

### The functions of PSK under different stresses

PSK peptides have been found to modulate plant responses to biotic or abiotic stresses in various plant species. Notably, *Arabidopsis* seedlings subjected to heat stress exhibit early senescence and reduced fresh weight, which can be alleviated by exogenously applying high concentrations of PSK peptides *in vitro* (Yamakawa et al., 1999). Additionally, the external application of PSK peptides can alleviate chilling injury symptoms, such as weight loss and internal browning in loquat (*Eriobotrya japonica* Lindl.) fruit under cold stress conditions (Song et al., 2017). Previously, it has been shown that osmotic stress significantly upregulates the expression of *proPSK1* and enhances downstream SBT3.8-mediated posttranslational processing, which results in the production of active PSK peptide to alleviate this stress (Stuhrwohldt et al., 2021). Furthermore, the ectopic expression of *OsPSKR15* enhances *Arabidopsis*’ sensitivity to ABA during germination, growth, and stomatal closure processes while improving drought resistance (Nagar et al., 2022). In particular, the kinase domain in OsPSKR15 also interacts with the ABA receptors, AtPYL9 and OsPYL11, to participate in ABA signal responses. Taken together, this indicates that PSK peptides play a role in facilitating plants stress responses to abiotic factors through an ABA-dependent mechanism (Nagar et al., 2022).

Under environmental stress, flowers and fruits are susceptible to premature abscission phenotype that results in reduced crop yield. In response to drought stress, the PSK signaling pathway has been shown to mediate premature abscission of floral organs in tomatoes (Reichardt et al., 2020). For example, the overexpression of *SlPhyt-2* (Phytaspase2, a subtilisin-like protease) in tomatoes leads to premature flower abscission, and drought stress was found to further increase the abscission rates up to 70% (Reichardt et al., 2020). Further studies also revealed that SlPhyt-2 cleaves the PSK precursor into mature PSK peptide and induces the expression of tomato abscission-related genes, *TAPG2* and *TAPG4* (Reichardt et al., 2020). Notably, this induction is independent of auxin or ethylene (Reichardt et al., 2020). Consequently, under drought stress, SlPhyt-2 promotes the generation of mature PSK peptides within the abscission zone and stimulates the expression of cell wall hydrolases to facilitate the detachment of floral organs (Reichardt et al., 2020) (**Figure 2A**).

### PSK receptors

*Arabidopsis* PSK peptide receptors (PSKRs) belong to the Leucine-rich repeat receptor kinase (LRR-RK) family. This kinase structure is composed of an N-terminal hydrophobic signal sequence, an extracellular repeat sequence that contains 21 leucine-rich repeats (LRRs), an island domain of 36 amino acids that serves as the binding site for PSK peptide, a transmembrane domain (TM), and a cytoplasmic kinase domain (Matsubayashi et al., 2002, 2006). Historically, PSKR1 and PSKR2 receptors have been identified through genetic screening in *Arabidopsis*, where PSKR1 plays a predominant role in growth and development compared with PSKR2 (Matsubayashi et al., 2002; Amano et al., 2007; Kutschmar et al., 2009).

PSKR1 possesses a guanylate cyclase (GC) catalytic center within its kinase domain that promotes activity both *in vivo* and *in vitro* (Kwezi et al., 2011). Irving’s group found that leaf protoplasts treated with PSK-α or an overexpression of *PSKR1* induces a rapid increase in cGMP levels (Kwezi et al., 2011). Likewise, a mutation in the G923 site (G923K) in PSKR1 reduces cGMP synthesis and fails to restore the short root phenotype in *pskr1-3pskr2-1* double knockout mutants (Ladwig et al., 2015), which highlights the crucial role of this site for PSKR1 function.

Additionally, PSKR1 undergoes auto- and transphosphorylation; however, the substitution of the conserved lysine (K762) within its ATP-binding region with glutamate significantly inhibits its kinase activity (Hartmann et al., 2014; Kaufmann et al., 2017). Previously, several studies have demonstrated that increasing the concentration of free calcium ions inhibits PSKR1 kinase activity, yet also enhances its guanylate cyclase activity. Together, this suggests that changes in calcium ion levels may serve as a direct molecular switch to modify signaling networks mediated by PSKR1 in plant growth, development, and stress responses (Muleya et al., 2014). Likewise, the interaction between CaM2 and hypophosphorylated PSKR1 is Ca^2+^-dependent. However, the binding of Ca^2+^-CaM2 does not influence its kinase activity (Kaufmann et al., 2017). Also, a mutation at the calmodulin (CaM)-binding kinase domain in PSKR1 (W831S) shows dephosphorylation activity *in vitro*. Ultimately, this impairs the binding ability of PSKR1 to CaM, which fails to rescue the growth defect of roots and rosette leaves in the *pskr1* mutant (Hartmann et al., 2014; Kaufmann et al., 2017).

**Figure 3:**
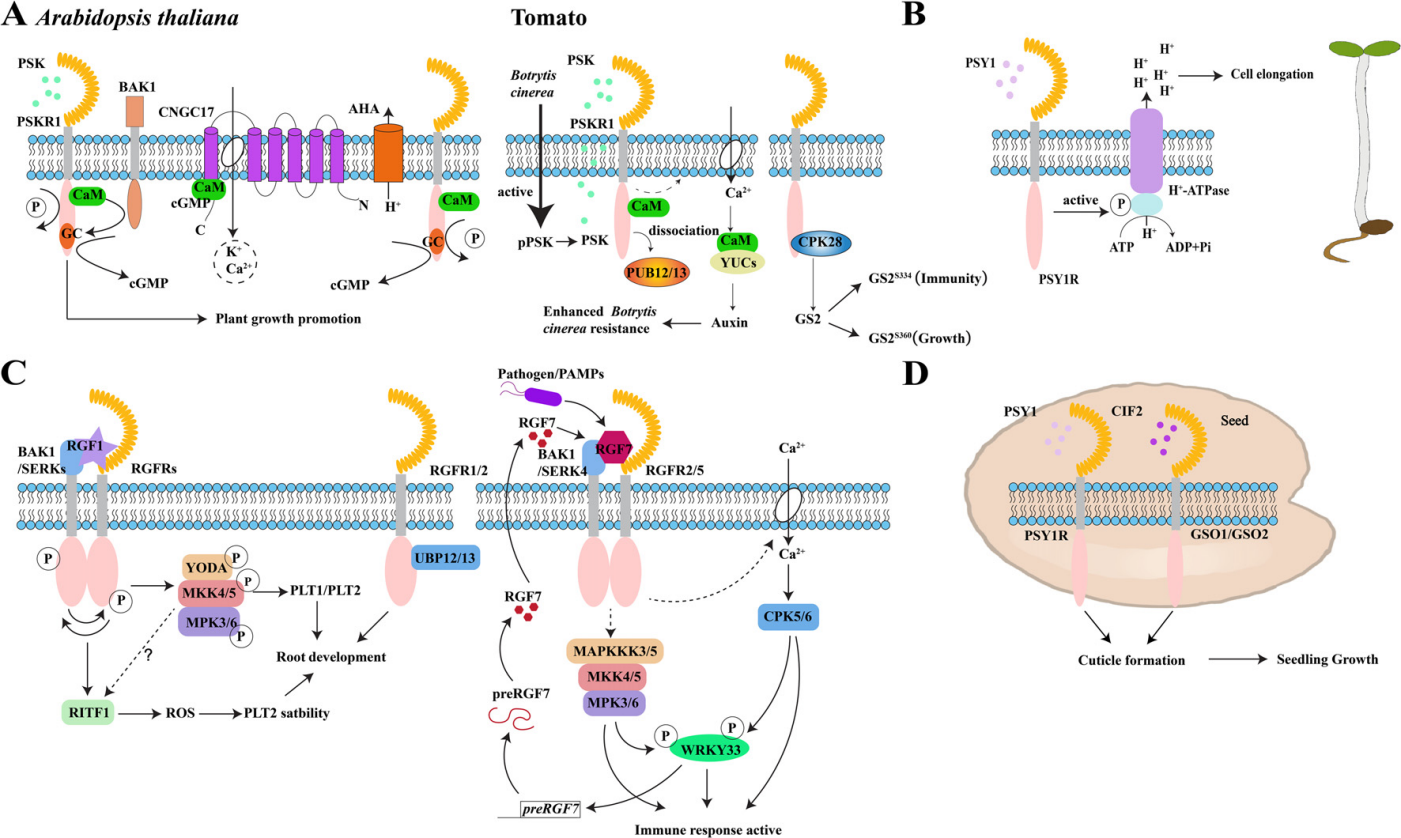
Peptide signaling mechanisms in plant growth and immunity. **(A)** PSK-PSKR signaling module to promote growth and disease resistance processes in *Arabidopsis* and tomatoes. pPSK, phytosulfokine precursor; PSKR1, PSK receptor, **(B)** PSY1 peptide binds to the receptor PSY1R to activate the protein kinase domain of PSY1R, which then activates the H^+^-ATPase regulatory domain on the plasma membrane by phosphorylation. Extracellular space acidification after proton exclusion promotes the elongation of *Arabidopsis* hypocotyls. **(C)** Signaling modules for RGF peptides and RGFR receptors and their co-receptors SERKs and BAK1 to regulate root meristem development and immune response in *Arabidopsis*. RGFR, RGF receptor; PAMPs, pathogen-associated molecular patterns; *preRGF7*, RGF7 precursor-encoding gene; **(D)** PSY1-PSY1R and CIF2-GSO1/GSO2 signaling regulates the formation of *Arabidopsis* embryonic cuticle to promote seedling growth. The schematic views in A, B, and C were modified from Ladwig et al., 2015; Fuglsang et al., 2014; Lu et al., 2020 and Wang et al., 2021b.

The CYCLIC NUCLEOTIDE-GATED CHANNEL17 (CNGC17) belongs to subgroup III in the CNGC family (Ladwig et al., 2015). Previously, Sauter’s group shows that root length in *atcngc17* mutants was significantly shorter than in the wild type and that this phenotype can be partially restored by PSK treatments (Ladwig et al., 2015). Their analysis also revealed that AtPSKR1 interacts with BAK1, as well as the H^+^-ATPases, AHA1, and AHA2. Together, these three proteins then bind to CNGC17, which is regulated by cGMP and CaM (Ladwig et al., 2015). They also determined that the increase in cGMP levels produced by AtPSKR1 activates the expression of *CNGC17*, which may form a functional core module to mediate cell expansion and regulate plant growth and development (Ladwig et al., 2015) (**Figure 3A**). Therefore, the interaction between Ca^2+^/CaM and PSKR1 is a pivotal point in the PSK signaling pathway.

By analyzing the crystal structure of the PSK-PSKR1^LRR^ complex, it was determined that PSK adopts a β-strand conformation to form an anti-parallel β-sheet structure with PSKR1^ID^ (the island domain of PSKR1) (Wang et al., 2015b). From this structure, it was found that somatic embryogenesis receptor-like kinases (SERKs) function as co-receptors with PSKR receptors (Wang et al., 2015b). Altogether, this allowed for the structure of the PSK-PSKR^LRR^-SERK^LRR^ activation complex to be resolved (Wang et al., 2015b). Under the influence of PSK peptides, this complex induces a novel interface between the initially disordered receptor island region and the co-receptor SERK. This binding then allosterically activates the PSKR receptor (Wang et al., 2015b).

Historically, SERK proteins regulate growth-defense trade-offs and serve as pivotal factors that influence the activity of plant development and immunity receptors. For example, in a recent study, a PSKR1 known as ALR1 was found to function as a plant Al ion sensor (Ding et al., 2024). The authors found that the cytoplasmic domain of ALR1 can bind to Al ions and recruit the BAK1 co-receptor, which induces ALR1-mediated phosphorylation of RbohD to boost the generation of reactive oxygen species (ROS). The production of ROS, in turn, enhances the oxidative modification of RAE1 F-box protein to impede the proteolysis of SENSITIVE TO PROTON TOXICITY 1 (STOP1, a zinc finger transcription factor). This sequence then detoxifies Al from the system by expelling organic acid anions. Ultimately, the dual functions of PSKR1 in PSK and Al ion sensing suggest that it may play a part in the interaction between plant growth and environmental stresses. Therefore, resolving the structure of the PSK receptor contributes to the design of commercial PSK analogues, which will serve as production additives to enhance crop yield and reduce stress response in agriculture.

### PSKs in plant immunity

In response to plant pathogens, it has been shown that the *pskr1* mutant exhibits different levels of disease resistance against biotrophic and necrotrophic pathogens (Mosher et al., 2013). Specifically, Kemmerling’s group found that a *pskr1* mutant shows enhanced immunity to *Pseudomonas syringae* pv. tomato DC3000 (*Pst* DC3000) but reduced immunity to the fungal pathogen *Alternaria brassicicola* (Mosher et al., 2013). This phenomenon suggests that cross-talk may exist between the SA and JA signaling pathways and that PSKR1 could modulate plant immune responses depending on its interaction with different phytohormones (Mosher et al., 2013). For instance, the flg22 peptide derived from bacteria is recognized as a microbe-associated molecular pattern (MAMP) that is capable of triggering pattern-triggered immunity (PTI) in *Arabidopsis* (Igarashi et al., 2012). Katagiri’s group found that *atpskr1* seedlings treated with the flg22 peptide decreased biomass and produced shorter root length compared with the wild type (Igarashi et al., 2012).

The influence of PSKs on immunity also exists in other plant species. OsPSKR1, located on the plasma membrane in rice, functions as a receptor for PSK and enhances rice resistance to *Xanthomonas oryzae* pv. *oryzicola* (*Xoc*) by activating the expression of resistance-related genes involved in the SA pathway (Yang et al., 2019) (**Table 2**). Previously, long non-coding RNAs (IncRNAs) have been identified as regulators of pathogen resistance in plants (Wang et al., 2014; Zhang et al., 2014; Wang et al., 2021a). For example, *GbIncRNA7* encodes a 74-aa PSK precursor peptide, and it has been shown that PSK-α content increases in *GbIncRNA7* overexpression lines (Zhang et al., 2022) (**Table 2**). The authors found that PSK-α treatment reduces the accumulation of *IncRNA7*, yet also increases the accumulation of IAA in cotton and activates *Pectin methylesterase inhibitor 13* (*GbPMEI13)* expression, which promotes pectin methylation and enhances resistance to *Verticillium dahliae* (Zhang et al., 2022). Furthermore, a CRISPR/Cas9-generated *Clpsk1* mutant conferred increased resistance against *Fusarium oxysporum* f.sp*. niveum* (*FON*) infection in watermelon [*Citrullus lanatus* (Thunb.) Matsum and Nakai] (Zhang et al., 2020), which suggests the potential role of PSKs in crop improvement.

In addition to bacteria, the necrotrophic fungus, *Botrytis cinerea* (*B. cinerea*), activates the production of mature PSK peptide in tomatoes, which is recognized by the SlPSKR1 receptor and leads to an elevation of cytosolic Ca^2+^ levels (Zhang et al., 2018). This transient increase in Ca^2+^ facilitates the binding of SlCaM to SlYUCs (auxin biosynthetic proteins) that activates downstream auxin biosynthesis and signaling transduction pathways that result in plant resistance against *B. cinerea* (Zhang et al., 2018) (**Figure 3A** and **Table 2**).

Additionally, the ubiquitin ligases SlPUB12 and SlPUB13 interact with SlPSKR1 and mediate its ubiquitination in tomatoes (Hu et al., 2023). Upon binding to the PSK, the ubiquitination of SlPSKR1 by SlPUB12/13 is inhibited, which results in the enhanced stability of the SlPSKR1 protein and stronger resistance against *B. cinerea* infection (Hu et al., 2023). Shi’s group showed a tomato mutant lacking calcium-dependent protein kinase *slcpk28* inhibited growth and increased resistance to *Pst* DC3000 (Ding et al., 2023). More specifically, they found that the interaction between SlGS2 and SlCPK28 kinase was enhanced by PSK peptide stimulation and resulted in phosphorylation at the 334th and 360th serine sites on the glutamine synthetase GS2 protein (Ding et al., 2023). Interestingly, these two sites elicit distinct regulatory responses in which GS2^S334^ regulates plant defense response, whereas GS2^360^ influences growth processes, which ultimately creates a trade-off between plant growth and defense through modulation by PSK peptides (Ding et al., 2023) (**Figure 3A**). However, the negative regulation role of PSKR1 in plant autoimmunity has also been established. In particular, it can be induced by *Pseudomonas* to suppress downstream SA immune signaling while also promoting *Pseudomonas* growth in the rhizosphere (Song et al., 2023). Taken together, these findings reveal that PSKR1 is essential to maintaining plant growth and restrain excessive defense responses during *Pseudomonas* treatment (Song et al., 2023).

In summary, these findings demonstrate the potential agricultural applications of PSK peptides in crop improvement. Deciphering the interplay between growth and immunity regulated by PSK is important to understand how plants effectively coordinate and optimize growth and defense under diverse environmental conditions.

## PSYs

### The precursors of PSY peptides

The PSY1 peptide consists of 18 amino acids and is known to promote cell proliferation at nanomolar concentrations (Amano et al., 2007). There are eight homologous genes for *PSY1* in *Arabidopsis*, and each is composed of approximately 90 amino acids and also features a conserved domain at the C-terminus (Tost et al., 2021; Ogawa-Ohnishi et al., 2022) (**Table 1** and **Figure 1B**).

Previously, it was shown that PSY precursor genes can be spatially expressed in various tissues of *Arabidopsis* (Ogawa-Ohnishi et al., 2022). In particular, *PSY1*, *PSY3*, and *PSY8* exhibit tissue-specific expression patterns throughout the plants. However, *PSY2* and *PSY5* are mainly expressed in shoot tissues, whereas *PSY4* and *PSY6* are primarily expressed in root tissues, and *PSY9* is expressed in inflorescences, siliques, and seeds (Ogawa-Ohnishi et al., 2022). Ultimately, the different spatial distribution of these PSY precursors in various tissues suggests that they play distinct roles in plants.

The PSY1 peptide is the most extensively investigated form of PSY peptides. Its active form undergoes two distinct posttranslational modification processes, namely, tyrosine sulfation and hydroxyproline arabinosylation, which result in the maturation of the peptide molecule (Amano et al., 2007). This mature peptide molecule plays a crucial role in regulating cell growth by activating the expression of genes involved in cell wall modification, thereby facilitating cell wall loosening (Mahmood et al., 2014).

Among the precursors *PSY1* to *PSY8*, *PSY1* and *PSY*4 are the primary members responsive to biotic stress (Tost et al., 2021). *PSY4* can be induced by *Colletotrichum tofieldiae* (*Ct*) and *Pseudomonas syringae* (*P. syringae*) (Tost et al., 2021). *Ct* is a non-pathogenic fungus that maintains normal plant growth under phosphorus-deficient conditions, suggesting a potential cooperation role between PSY4 and *Ct* in promoting root growth under nutrient-deficient conditions (Tost et al., 2021). Conversely, PSY1 is specifically induced by *P. syringae* and plays a key role in the plant immune response against bacterial infection (Tost et al., 2021).

### The functions of PSYs in plant growth

Under the treatment of PSY5 peptide, the downregulated differentially expressed genes in the *tpst* mutant were primarily enriched in stress response mechanisms, including oxidative stress, SA, and JA signaling pathway, as well as cold and pathogen-induced stress responses (Ogawa-Ohnishi et al., 2022). These results indicate the pivotal role of PSY peptides in fine-tuning the trade-off between plant growth and stress response.

Recently, three LRR-RKs, namely, PSYR1, PSYR2, and PSYR3, were identified in *Arabidopsis*. The PSY-PSYR signaling pathway is crucial for maintaining the trade-off between plant growth and stress responses. Under stressful conditions, plants activate a protective response by attenuating the interaction between PSYRs and PSYs (Amano et al., 2007; Ogawa-Ohnishi et al., 2022). Pathogen invasion increases cell damage, decreasing the concentration of PSY peptides at adjacent sites and triggering a stress response around the site of damage (Ogawa-Ohnishi et al., 2022). The *psyr1psyr2psyr3* triple mutant exhibits slightly longer root length than the wild type due to increased cortical root cell length (Ogawa-Ohnishi et al., 2022). Furthermore, the *psyr1psyr2psyr3* mutant is more sensitive to salt stress, high temperature, and *P. syringae* infection compared with the wild type, suggesting that PSYRs may play a positive role in plant responses to various environmental stresses (Ogawa-Ohnishi et al., 2022).

Categorically, *PSY1R* (At1g72300) belongs to the LRR-RK family of receptor kinases and is a paralogous gene for the PSKR1 and PSKR2 receptors (Amano et al., 2007). Previous studies have long considered it as a receptor for the PSY1 peptide (Amano et al., 2007). However, recent research has shown that PSY1R is not a direct ligand-sensing receptor for the PSY peptide (Ogawa-Ohnishi et al., 2022). Nevertheless, it plays a crucial role in regulating plant growth, development, and immune response through PSY peptide signaling. In particular, a *pskr1pskr2psy1r* triple mutant was found to exhibit dwarfism and reduced cell number and size (Amano et al., 2007). Consistently, the *psy1r* mutant has also been found to display a shorter hypocotyl phenotype compared with a wild type due to decreased cell length (Fuglsang et al., 2014).

Additionally, autophosphorylation is observed with the PSY1R receptor kinase, primarily at amino acid K831, and it has been determined that ATP supplementation enhances this activity (Oehlenschlaeger et al., 2017). Likewise, a mutation in the lysine residue (K831A) within the cytosolic kinase domain of PSY1R (kPSY1R) abolished this autophosphorylation activity (Oehlenschlaeger et al., 2017). Like PSKR1, PSY1R can interact with SERK family proteins, and kPSY1R serves as a target for SERK kinases to activate signaling transduction (Amano et al., 2007; Wang et al., 2015b; Oehlenschlaeger et al., 2017) (**Figure 2B**).

PSY1R also interacts with the plasma membrane proton pumps, AHA1 and AHA2. Its intracellular protein kinase domain phosphorylates the Thr881 site in the autoinhibitory region of the AHA2 C-terminal domain, which enhances its activity (Fuglsang et al., 2014). Consequently, PSY1 peptide treatment induced rapid phosphorylation of the Thr881 site and promoted proton efflux from roots, which ultimately suggests that the PSY1-PSY1R module promotes plant growth by activating H^+^-ATPase on the plasma membrane (Fuglsang et al., 2014) (**Figure 3B**).

### The functions of PSYs in plant immunity

PSYR1, PSYR2, and PSYR3 receptors are capable of recognizing nine PSY peptides, as well as the sulfated peptide RaxX (XA21-mediated immunity X) (Amano et al., 2007; Ogawa-Ohnishi et al., 2022). Historically, RaxX is an analog synthesized by the biotrophic pathogen *Xanthomonas oryzae pv. oryzae* (Xoo) that can enhance root elongation in *Arabidopsis* and rice (Pruitt et al., 2015, 2017). However, its receptor, XA21, specifically recognizes and regulates plant immunity processes and is not activated by the PSY1 peptide (Pruitt et al., 2015, 2017).

The PSY1-PSY1R signaling pathway exerts an antagonistic effect on plant defenses against biotrophic and necrotic pathogens, similar to the antagonism observed in the PSK-PSKR1 signaling pathway during plant immune responses to diverse pathogens (Igarashi et al., 2012; Mosher et al., 2013). Kemmerling’s group found that a *pskr1psy1r* double mutant showed more severe disease resistance than the single mutant (Mosher et al., 2013). Similar to the PSK peptide, PSY1 induces susceptibility in *Arabidopsis* plants to *Fusarium oxysporum* fungi (Shen and Diener, 2013), which may be attributed to the ability of *Fusarium oxysporum* to inhibit negative feedback regulation of PSY1R that stabilizes the PSY1 signaling pathway (Shen and Diener, 2013). Taken together, these findings highlight the crucial roles of PSY in maintaining a trade-off between plant growth and immunity.

## RGFs

RGF peptides, also known as GOLVEN (GLV) or CLE-like (CLEL) peptides, are a class of plant-specific peptide hormones that are widely distributed in planta (Whitford et al., 2012; Fang et al., 2021). Typically, RGF/GLV/CLEL peptide precursors consist of approximately 100 amino acids that are modified to form mature peptides through tyrosine sulfation and proteolytic processes (Matsubayashi, 2012) (**Table 1** and **Figure 1C**). There are 13 precursors for the RGF/GLV/CLEL peptide family in *Arabidopsis* (Matsuzaki et al., 2010; Meng et al., 2012; Whitford et al., 2012). Of which, nine RGF peptide precursors (RGF1–9) were identified based on their sequence characteristics that include a secretory signal and a conserved domain of Asp-Tyr (DY) at the C-terminus (Matsuzaki et al., 2010).

Further screening has also revealed three additional RGF peptides named RGF10, GLV8, and GLV9 in *Arabidopsis* (Whitford et al., 2012; Shinohara, 2021). Notably, CLE18 exhibits distinct features compared with other members of the CLE peptide precursors. The CLE motif in CLE18 is situated in the variable region rather than at the C-terminus, and overexpression of this gene promotes root elongation (Meng et al., 2012). The C-terminal region of the CLE18 precursor contains a 13-amino acid motif, which suggests its classification within the CLEL family that encodes for RGF peptides through homology comparison analysis (Meng et al., 2012). However, unlike other RGF peptides, unmodified CLE18 possesses activity that alters root growth direction and regulates lateral root development (Meng et al., 2012). Additionally, other investigations into the RGF/GLV/CLE peptide family also revealed that SBT6.1 and SBT6.2 play essential roles in cleaving the RGF6/CLEL6/GLV1 precursor (Ghorbani et al., 2016).

### The function of RGFs in plant growth

RGF peptides play a conserved role in promoting root meristem formation across diverse plant species (Fang et al., 2021). This peptide family modulates the maintenance of the root meristem, the development of root hairs, and hypocotyl geotropism (Fernandez et al., 2013) and sustains cell proliferation activity in the root tip meristem (Shinohara, 2021).

Of these, *RGF1* is predominantly expressed in the quiescent center and the columella stem cells, whereas *RGF2* and *RGF3* are primarily expressed in the innermost layer of central columella cells (Matsuzaki et al., 2010; Matsubayashi, 2011). Ultimately, the RGF1 peptide is crucial for maintaining meristem and root growth (Matsuzaki et al., 2010), and it has been shown that the elevation of *RGF1* expression or supplementation with the bioactive form of RGF1 peptide leads to an increase in *Arabidopsis* root meristem activity. This was also consistent with a *rgf1rgf2rgf3* triple mutant that exhibited a reduced root length phenotype (Matsuzaki et al., 2010).

RGF4/6/9 play a crucial part in root geotropism by regulating auxin distribution, which controls root growth, lateral root development, and sensitivity to phosphorus deficiency conditions (Meng et al., 2012; Cederholm and Benfey, 2015; Fernandez et al., 2015). The effects of different RGF peptides on plant growth and development vary significantly. Overexpression of *GLV6/RGF8/CLEL2* leads to a reduction in lateral roots and disruption of the first asymmetric cell division during primordium formation (Fernandez et al., 2015). Typically, RGF1 and RGF2 peptides impact various aspects of *Arabidopsis* root development under phosphate deficiency conditions, including that RGF1 primarily affects the number of circumferential cells in root meristem, whereas RGF2 influences the longitudinal growth rate of the primary root (Cederholm and Benfey, 2015). *RGF6/GLV1* overexpression lines exhibit distinct phenotypes, including gravity-curled roots and elongated hypocotyl in *Arabidopsis* (Whitford et al., 2012; Ghorbani et al., 2016), which can be restored to the wild-type phenotypes by mutating either *SBT6.1* or *SBT6.2* genes (Ghorbani et al., 2016). The Sperpin 1 protease inhibitor binds to SBT6.1 and inhibits its activity, thereby suppressing the activity of RGF6/GLV1 peptide and hypocotyl elongation (Ghorbani et al., 2016).

### RGF receptors

RGFR1/RGI3 (AT4g26540) belongs to the LRR-RK family and acts as a receptor for the RGF1 peptide (Song et al., 2016). Previous crystal structure analysis of the RGFR1-RGF1 complex revealed that the specific recognition between these two molecules is primarily mediated by the Arg-x-Gly-Gly (RxGG) motif (Song et al., 2016). From this work, four additional LRR-RKs were identified based on this conserved motif, namely, RGFR2/RGI4 (At5g56040), RGFR3/RGI2 (At5g48940), RGFR4/RGI1 (At3g24240), and RGFR5/RGI5 (At1g34110), which are all capable of recognizing RGF peptides (Ou et al., 2016; Song et al., 2016).

Given the diverse nature of RGF peptide signaling, it is likely that different RGFR receptors mediate specific recognition events to regulate distinct downstream targets involved in plant growth and development. Notably, mutants such as *serk1serk2* and *serk1serk2bak1* show reduced meristem size and shorter roots similar to those observed in *rgfr* and *rgf1/2/3* mutants (Song et al., 2016). Further biochemical investigations have also demonstrated that BAK1 and SERKs function as co-receptors for RGFRs in the RGF-induced signal transduction pathway that governs root meristem development (Song et al., 2016).

The five RGFR receptors all interact with BAK1, and this interaction depends on the presence of the RGF1 peptide (Ou et al., 2022). Likewise, it has been shown that phosphorylation levels of RGFR1, RGFR3, RGFR4, and BAK1 significantly increase with RGF1 peptide treatment. However, the phosphorylation status of RGFR2, RGFR5, SERK1, and SERK2 is not unaffected (Ou et al., 2022). By examining the phosphorylation status of RGFR4 in both wild-type and *serk1-8bak1-4* double mutant backgrounds, Li’s group found that RGF1 treatment significantly enhances the phosphorylation status of RGFR4 in wild-type plants but does not induce any significant changes in the mutant background (Ou et al., 2022). Additionally, RGF1 induces BAK1 protein phosphorylation in wild-type plants that also does not alter the phosphorylation level of the BAK1 protein in a *rgfr12345* mutant. Together, these data indicate that RGF1 induces interdependent phosphorylation of both RGFRs and BAK1 (Ou et al., 2022). Additionally, their experiments also revealed a reciprocal transphosphorylation between RGFR4 and BAK1, suggesting that the extracellular kinase domain of RGFR4 can phosphorylate the extracellular kinase domain of BAK1 and vice versa (Ou et al., 2022) (**Figure 3C**).

### The signaling pathway of RGFs-RGFR in plant growth

The lateral root density of the *glv6glv10* (*rgf8/rgf5*) double mutant is significantly increased compared with the wild type (Fernandez et al., 2020). Meanwhile, GLV6/RGF8 is capable of inducing the phosphorylation and activation of MPK6 (Fernandez et al., 2020), suggesting that MPK6 functions as a downstream effector in the regulation of auxin-mediated lateral root development through the GLV6/10(RGF8/5)-RGFR2/4/5 signaling pathway (Fernandez et al., 2020) (**Figure 2C** and **Table 2**). Consistently, exogenous application of the RGF1 peptide significantly upregulates *PUCHI* expression and inhibits lateral root development (Jeon et al., 2022). The insensitive phenotypes observed in *rgfr4*, *puchi1*, and *mpk6* mutants upon RGF1 treatment further confirm that the RGF1-RGFR4 module negatively regulates lateral root development in *Arabidopsis* by activating *PUCHI* expression via MPK6 (Jeon et al., 2022).

With regard to signaling, RGF1 activates the mitogen-activated protein kinase (MAPK) signaling cascade of the RGFRs-dependent YODA (MAPKKK)-MKK4/MKK5-MPK3/MPK6 pathway to promote the expression of downstream *PLT1*/*PLT2* genes and regulate root growth and development through the RGF1-RGFR module (Lu et al., 2020; Shao et al., 2020) (**Figure 3C**). In particular, the activation of this signaling cascade by RGF1 depends on RGFRs and SERKs (Ou et al., 2022).

Typically, RGF1 interacts with RGFR4, which results in the phosphorylation and ubiquitination of RGFR4 (Ou et al., 2016). The authors also found that the *ubp12,13* double mutant lacks ubiquitin-specific proteases UBP12 and UBP13 that results in shorter roots and fewer cortical root meristem cells compared with the wild type that also exhibits insensitivity to RGF1 treatment (Ou et al., 2016; An et al., 2018). Here, they found that overexpressing *PLT2* or *RGFR1* can partially restore the growth defect in the *ubp12,13* mutant (An et al., 2018). Furthermore, UBP13 directly interacts with both RGFR1 and RGFR2, and its overexpression increases the abundance of the RGFR1 protein even in the absence of RGF1 peptide by inhibiting the ubiquitination degradation induced by RGF1 (An et al., 2018). Ultimately, these findings indicate that UBP13 regulates the deubiquitination of the RGFR1 receptor in *Arabidopsis* to maintain the stability of the RGFR1 protein in plants for proper root meristem development (An et al., 2018) (**Figure 3C**).

The RITF1 protein, a member of the PLANT AT-RICH SEQUENCE AND ZINC-BINDING TRANSCRIPTION FACTOR (PARTZ) family, was identified through screening for genes induced by the RGF1 peptide (Yamada et al., 2020). RITF1 can expand the root meristem zone, increase cell numbers, and significantly enhance O^2−^ signals, consistent with the root phenotype observed after RGF1 peptide treatment (Yamada et al., 2020). Overexpression of *RITF1* in the *rgfr1,2,3* triple mutant restores its defective root development phenotype, indicating that RITF1 regulates ROS levels and distribution in *Arabidopsis* roots (Yamada et al., 2020). These studies elucidate a mechanism by which RGF1 induces the expression of *RITF1* in the meristem to regulate ROS levels and distribution, thereby modulating *Arabidopsis* root development via the PLT2 protein (Yamada et al., 2020) (**Figure 3C**). Both the RGF1-RITF1-PLT2 module and the YODA-MKK4/MKK5-MPK3/MPK6 signaling pathway play roles in regulating root development in *Arabidopsis*. However, the relationship between these two signaling pathways remains to be investigated.

### The function of RGFs in plant immunity

Previously, it was found that the expression of the *RGF6/GLV1* and *RGF9/GLV2* genes in leaves was significantly downregulated upon infection with *Pseudomonas syringae*, whereas overexpression of these two genes exhibits a pronounced disease resistance phenotype (Stegmann et al., 2022), which suggests their involvement in plant immunity. The authors found that pretreatment of plant leaves with the RGF9/GLV2 peptide enhances their sensitivity to flg22 (Stegmann et al., 2022). Likewise, the cotreatment of plants with the RGF9/GLV2 peptide and flg22 increased ethylene production and *PR1* gene expression and reduced fresh weight, which indicates that the RGF9/GLV2 peptide plays a crucial role in regulating the intensity of the PTI immune response triggered by flg22 (Stegmann et al., 2022) (**Table 2**). The authors also showed that the interaction between RGF9/GLV2 and RGFR1 promotes FLS2 protein abundance (Stegmann et al., 2022). Moreover, treatments with flg22 or GLV2 peptide enhance interaction between RGFR1 and FLS2 (Stegmann et al., 2022). They also found that *Arabidopsis* seedlings treated with GLV2 peptide also facilitated the formation of the FLS2-BAK1 complex, which provides valuable insights into how the RGF-RGFR signaling pathway regulates the PTI immune response (Stegmann et al., 2022).

Among the precursors for *preRGF1-11*, only *preRGF7* can be significantly induced by *P. syringae*. Transgenic plants overexpressing *preRGF7* exhibit enhanced defense responses and increased resistance to pathogens, positioning it as an endogenous activator of the plant immune response (Wang et al., 2021b). Endogenously synthesized mature RGF7 peptides bind to RGFR2/RGFR5 receptors, along with BAK1 and SERK4 co-receptors, activating downstream immune signal in *Arabidopsis* to maintain normal plant growth (Wang et al., 2021b). Additionally, RGF7 induces the activation of downstream MPK3 and MPK6 kinases, promoting *preRGF7* expression (Wang et al., 2021b). WRKY33 directly binds to the promoter of *preRGF7*, inducing its expression in response to pathogen attack (Wang et al., 2021b). Furthermore, WRKY33 acts as a phosphorylation substrate for MPK3/MPK6 and CPK5/CPK6 protein kinases, which synergistically activate its transcriptional activity (Zhou et al., 2020). After pathogen treatment, CPK5/CPK6 and MPK3/MPK6 upregulate *WRKY33* expression through a positive feedback loop mediated by RGF7 (Wang et al., 2021b) (**Figure 3C**).

The extracellular pH of plants is 5.7 in an acidic form. However, upon elicitation of the plant immune response, it becomes alkaline, which is essential for root tip meristem (RAM) growth mediated by RGF1 (C Grignon and Sentenac, 1991; Geilfus, 2017; Liu et al., 2022). Extracellular alkalization is known to impede the RGF1 signaling pathway. In particular, Pep1 peptide (a DAMP molecule) treatment or pathogen-associated molecular pattern (PAMP) can induce extracellular alkalization of the root apical meristem to inhibit its growth (Liu et al., 2022). In this mechanism, a sulfite group from a tyrosine residue exists in the RGF1 peptide (sY2), and under acidic conditions, sY2 becomes protonated to promote a strong hydrogen bond interaction between the RGF1 peptide and its receptor through the RxGG motif (Liu et al., 2022). Gradually, extracellular alkalization hinders the acid-dependent interaction between RGF1 and its receptor RGFRs via pH sensor sY2 (Liu et al., 2022). However, extracellular alkalization also facilitates base-dependent binding between plant elicitor peptides (Peps) and their receptor PEPRs through a pH sensor, Glu/Asp, to promote immunity (Liu et al., 2022). Additionally, this exchange of extracellular domains between RGFR and PEPR alters the pH dependence of RAM growth (Liu et al., 2022). Ultimately, this mechanism elucidates how plant peptide–receptor complexes sense extracellular pH to regulate RAM growth and immunity and provides novel insights into regulating crop development and stress response through acid–base regulation.

## CIFs

### The precursors of CIF peptides

Five precursors, known as CASPARIAN STRIP INTEGRITY FACTORs (CIF1–4) and TWISTED SEED (TWS1), are involved in the synthesis of CIF peptides in *Arabidopsis* (Nakayama et al., 2017; Doll et al., 2020). The pro-peptides encoded by these genes undergo processing to form mature peptides that contain 21–24 amino acids (Fujita, 2021) (**Table 1** and **Figure 1D**). Similar to the other three sulfated peptides, the CIF peptide precursors contain a conserved domain near the C-terminus that facilitates the production of functional peptide hormones through posttranslational modification and proteolysis processes (Nakayama et al., 2017). Additionally, the pollen-specific subtilisin, SBT5.4, cleaves the C-terminal extension of the CIF4 precursor and its active peptides to play an important role in regulating tapetum activity (Truskina et al., 2022). Specifically, TWS1 interacts with subtilisin SBT1.8 and recognizes tyrosine sulfation as a posttranslational modification for the TWS1 precursor, which ensures the activity of the TWS1 peptide to promote growth and development (Royek et al., 2022).

DY is a conserved N-terminal sulfation motif in mature CIF peptides, as well as other sulfated peptides except for GLV9 (Amano et al., 2007; Matsuzaki et al., 2010; Kaufmann and Sauter, 2019). Apart from the DY motif, CIF family peptides also exhibit hydroxylation of two proline residues, which is conserved among this group (Nakayama et al., 2017). Proline hydroxylation is an important posttranslational modification catalyzed by prolyl-4-hydroxylase (P4Hs) in peptide biology (Kaufmann and Sauter, 2019). Additionally, hydroxyproline (Hyp) serves as a scaffold for the addition of pentose to PSY through Hyp O-arabinosyltransferase (HPAT), which is an essential modification for the biological activity of peptides (Amano et al., 2007; Ogawa-Ohnishi et al., 2013).

### The function of CIFs in Casparian strip development

The absorption of nutrients by roots is a fundamental process in plant growth and development. Mineral ions are primarily transported into epidermal cells through transporters that then pass through the endodermis via symplasts on the plasmodesmata that are finally secreted into the xylem for transportation to aboveground tissues. In vascular plants, nutrients are accumulated in the xylem vessels in an inverse concentration gradient. Consequently, a circular physical barrier called the Casparian strip (CS) evolves within the endoderm cell wall to prevent passive diffusion of ions and water through the endothelial cells that surround the vascular bundles (Geldner, 2013). As a physical barrier, the CS restricts the inward or outward leakage of unfavorable ions between xylem and soil (Robbins et al., 2014). Plants also employ a hydrophobic barrier on the endoderm to limit solute diffusion from the soil into the stele, which has significant implications for plant development (Geldner, 2013). Taken together, this mechanism facilitates efficient water and nutrient transport from roots that also provides defense against soil-borne pathogens.

Geldner’s and Belkhadir’s groups determined that a *tpst-1*/*sgn2-2* mutant exhibited defects in CS formation, as well as severe impairment in root growth (Doblas et al., 2017). The integrity factors, CIF1 and CIF2, which are peptide hormones closely associated with CS formation, have been identified in *Arabidopsis* Nakayama et al., 2017). Typically, *CIF1* and *CIF2* are expressed in the root stele and can interact with GASSHO1(GSO1)/SCHENGEN3(SGN3), which are LRR-RKs expressed in the endoderm, along with its homolog GSO2 (Nakayama et al., 2017). SGN1 is a receptor-like cytoplasmic kinase that also co-localizes with SGN3 at the outer edge of the CS domain (Doblas et al., 2017). In their work, the authors also found loss-of-function mutations in SGN1 and SGN3 that resulted in discontinuous CS formation that potentially form a local signal module with enhanced activity to regulate plant CS development (Doblas et al., 2017). SGN1 is typically located outside the plasma membrane from the endodermis to cortex, whereas CIF peptides reside within the inner cell layers (Doblas et al., 2017). However, activation of the SGN3 receptor by CIF peptides through the CIF-SGN3-SGN1 signaling pathway enables signal transmission through SGN1 without establishing an extracellular diffusion barrier for CS (Doblas et al., 2017) (**Figure 2D**). Matsubayashi’s group found that a *cif1,2* double mutant also exhibited sensitivity to iron and that high concentrations of iron significantly suppress growth (Nakayama et al., 2017). Additionally, both acidic and iron treatments can induce the expression of *CIF1* and *CIF2* genes. However, iron is more soluble under acidic soil conditions compared with neutral soils (Nakayama et al., 2017). Collectively, these findings suggest that CIF peptides assist plants to cope with adverse soil conditions to ensure normal survival and growth (Nakayama et al., 2017).

In addition, recent studies have revealed the regulatory role of CIF peptides in the gene regulatory network that governs CS formation in rice. Three homologous genes that encode CIFs (*OsCIF1a*, *OsCIF1b*, and *OsCIF2*) have been identified in rice (Zhang et al., 2024). Receptor-like kinases encoded by *OsSGN3a* and *OsSGN3b* act as receptors for these CIFs (Zhang et al., 2024). Notably, OsSGN3 is specifically localized in the CS of the endodermis, and mutations in either *OsCIFs* or *OsSGN3s* result in a discontinuous CS and the aberrant accumulation of lignin and suberin within the CS of the endodermis (Zhang et al., 2024). Previously, the authors found through their RNA-seq data that the expression of *OsSGN3b* and *OsSGN1s* is regulated by OsMYB36a/b/c transcription factors (Zhang et al., 2024). Likewise, the overexpression of *OsCIFs* significantly upregulates key regulatory genes involved in CS formation, such as SHORTROOT1 (*OsSHR1*), *OsSHR2*, SCARECROW (*OsSCRs*) (Zhang et al., 2024). In addition, *AtMYB36* can be activated by the AtSHR-AtSCR module (Kamiya et al., 2015; Liberman et al., 2015; Li et al., 2018; Reyt et al., 2020). Thus, the gene regulatory networks for OsCIF1/2-OsSGN3a/b and OsSHR-OsSCR-OsMYB36a/b/c have been extensively researched and shown to regulate the formation of CS in the endodermis and non-endodermis of rice, respectively (Zhang et al., 2024). These findings demonstrate the conserved functions of CIF1 and CIF2 in the formation of CS in *Arabidopsis* and rice, which indicates their potential role in regulating nutrient absorption selectivity and offers encouraging prospects for crop genetic enhancement.

### The functions of CIFs in development of embryonic cuticle and pollen grains

The formation of the *Arabidopsis* embryonic cuticle relies on two receptor-like kinases, GSO1 and GSO2, as well as the subtilisin protease, ALE1 (Creff et al., 2019). Previously, it has been shown in several studies that pleiotropic phenotypes are observed in a *gso1gso2* double mutant that creates ectopic adhesion between cotyledons, abnormal embryo bending, a highly permeable epidermis, and shortened hypocotyls (Tanaka et al., 2001; Tsuwamoto et al., 2008; Xing et al., 2013; Creff et al., 2019). Notably, it has also been found that *cif1,2* and *gso1gso2* double mutants exhibit obvious defects in endodermal barrier formation in roots (Nakayama et al., 2017). The authors also found that the CS formation defect for *cif1*,2 could be rescued by exogenous CIF1 peptide. Here, the cotyledons from the *cif1*,2 seedlings exhibited normal growth without displaying the ectopic adhesion observed in *gso1gso2* due to embryonic cuticle defects, which suggests that GSO1 and GSO2 act as receptors for CIF1 and CIF2 peptides (Nakayama et al., 2017). However, it is possible that there are other ligands that also bind to these receptors for their function (Nakayama et al., 2017). Hothorn’s and Geldner’s groups used a quantitative biochemical interaction screening with a CIF antagonist and gene analysis approaches that revealed that the SERK protein acts as an essential co-receptor kinase for proper function for GSO1/SGN3 and GSO2 receptor kinases (Okuda et al., 2020).

*Arabidopsis* TWISTED SEED1 (TWS1) encodes a sulfated peptide of 23 amino acids with similar functions to CIF1 and CIF2 peptides (Doll et al., 2020). TWS1 is localized in the endomembrane system upstream of the vacuole and is actively expressed throughout all stages of *Arabidopsis* growth, with the highest levels observed in seeds (Fiume et al., 2016). It plays a crucial role to induce ectopic endodermal lignification and significantly contributes to embryonic cuticle formation (Fiume et al., 2016; Doll et al., 2020; Royek et al., 2022). Previously, it was shown in several studies that *in vitro* synthesized sulfated TWS1 peptide treatment could restore the defective phenotype of the *cif1,2* double mutant by acting as a ligand for GSO1 and GSO2 receptors (Doll et al., 2020; Royek et al., 2022). Here, the *tws1* mutant displayed a pleiotropic phenotype with stunted growth, cup-shaped cotyledons, shorter hypocotyls and siliques, disrupted epidermal tissue structure, irregular cell surface morphology, altered seed morphology, and increased root hair density (Fiume et al., 2016). In addition, loss-of-function mutants enhanced the accumulation of starch, sucrose, and protein but decreased fatty acid content in seeds (Fiume et al., 2016) (**Figure 2D**). Overall, it is necessary to continue the investigation of TWS1’s function, since it is important for plant growth and development, as well as for the biosynthesis, transportation, and storage of seed nutrients.

The working mechanism of CIF peptides in embryonic cuticle formation was elucidated through investigations on GSO1/2 receptors and subtilisin protease ALE1 (Creff et al., 2019). *ALE1* is only expressed in the endosperm, where it activates the mature TWS1 peptide secreted from the embryo due to an incomplete cuticular barrier (Doll et al., 2020). The activated TWS1 peptide then traverses the cuticle gaps and binds to GSO1 and GSO2 receptors within the embryo to promote local gap repair (Doll et al., 2020). However, when the embryonic cuticle remains intact, the inactive TWS1 peptide is confined to the embryo (Doll et al., 2020). Together, these findings indicate that subtilisin can act as a bidirectional peptide that regulates embryonic cuticle development. Ultimately, it also emphasizes the primary functions of CIF1, CIF2, and GSO1 signaling pathways in CS formation (Doblas et al., 2017; Nakayama et al., 2017; Doll et al., 2020).

In addition, Ingram’s group found that pollen grains from a *gso1gso2* double mutant exhibit the formation of enlarged and fused aberration, whereas the individual single mutants display normal phenotypes during pollen development (Truskina et al., 2022). Similar defective phenotypes in pollen were also observed in a *cif3,4* double mutant, which indicates that CIF peptides regulate GSO1/2-dependent pollen development (Truskina et al., 2022). Furthermore, CIF3 and CIF4 are involved in tapetum development regulation by binding to CIF ligands through GSO receptors located in the middle layer to potentially induce the polarization of tapetum cells (Truskina et al., 2022). Altogether, this process facilitates the secretion of pollen wall components into the anther cavity for deposition onto developing pollen grains, which then coordinates tapetum function with proper pollen grain development (Truskina et al., 2022) (**Figure 2D**).

Ultimately, the transition from embryo to seedling development is not an autonomous process and depends on factors secreted by the endosperm. Indeed, the presence of mature endosperm enhances the development of the seedling cuticle (De Giorgi et al., 2021). For example, embryo-endosperm grafting experiments revealed that the endosperm of imbibed seeds produce sulfated CIF2 and PSY1 peptides that bind to GSO1/2 and PSY1 receptors on the epidermis to promote cuticle formation and facilitate normal plant growth (De Giorgi et al., 2021) (**Figure 3D**).

## Concluding remarks and future perspectives

The peptides in *Arabidopsis* involve the coding of over 7,000 genes and encompass 600 receptor-like kinases (RLKs), but the activation mechanisms underlying these peptides and their corresponding receptors remain largely unknown (Takahashi et al., 2019). The synthesis of mature peptides requires posttranslational modification and proteolysis of precursors. In particular, subtilisin proteins have been identified as key players in this pathway. For example, the AtPSK4 precursor requires SBT1.1 for its proteolysis processing (Srivastava et al., 2008), whereas SBT6.1 is essential for RALF23 peptide maturation (Srivastava et al., 2009). Nevertheless, there is still a lack of clarity in many aspects concerning proteolysis processes for peptide precursor proteins. While some initial research suggests that the subtilisin family may play a crucial role as key enzymes in the proteolysis process, further investigations into the function of the subtilisin family proteins is significantly important to understand their biological functions with peptides.

Initially, most peptide research focused on investigating their impact on plant growth and development, including processes such as cell elongation, callus proliferation, root meristem development, pollen tube elongation, and crop yield. However, subsequent studies gradually shifted toward the investigation of peptide functions in plant immunity. Clearly, most sulfated peptides regulate both plant growth and development, as well as plant immunity. Still, the regulatory mechanisms that govern the trade-offs between these two processes have yet to be fully deciphered. Therefore, future research should focus on elucidating the molecular mechanisms through which peptides regulate plant growth, development, and immunity.

PSK and PSY1 were initially identified as growth factors that promote cell expansion and plant growth. However, subsequent studies gradually revealed that these two peptides significantly attenuate the PTI immune response in plants, while also enhancing their sensitivity to biotrophic pathogens and resistance against necrotic pathogens (Mosher and Kemmerling, 2013; Mosher et al., 2013). RGFs are known to play a role in regulating lateral root initiation and development in *Arabidopsis* (Jeon et al., 2022). Specifically, previous work has shown that pretreatment with the RGF9/GLV2 peptide enhances plant sensitivity to flg22 (Fernandez et al., 2013; Stegmann et al., 2022), and that *P. syringae* induces *preRGF7* expression, activates the MAPK signaling cascade, and triggers downstream immune signal gene expression through WRKY33 transcription factor phosphorylation (Wang et al., 2021b). In comparison, CIFs primarily affect CS formation, embryonic cuticle development, and embryo/pollen development in *Arabidopsis* (Fiume et al., 2016; Nakayama et al., 2017; Okuda et al., 2020; Royek et al., 2022). However, whether this kind of peptide plays a role in plant immunity remains unclear.

Studies have demonstrated that peptides exhibit functional conservation across diverse plant species, suggesting the potential to develop economically valuable crops by harnessing the biological functions of different peptides. These functions can lead the creation of novel crop varieties characterized by vigorous growth, enhance disease resistance, and higher yield potential. As a result, small peptides are emerging as a research hotspot for both fundamental studies in plant biology and applied studies in fruit and crop improvement.
